# Evidence for Reverse Causality in the Association Between Blood Pressure and Cardiovascular Risk in Patients With Chronic Kidney Disease

**DOI:** 10.1161/HYPERTENSIONAHA.116.08386

**Published:** 2017-01-11

**Authors:** William Herrington, Natalie Staplin, Parminder K. Judge, Marion Mafham, Jonathan Emberson, Richard Haynes, David C. Wheeler, Robert Walker, Charlie Tomson, Larry Agodoa, Andrzej Wiecek, Sarah Lewington, Christina A. Reith, Martin J. Landray, Colin Baigent

**Affiliations:** From the Clinical Trial Service Unit and Epidemiological Studies Unit (CTSU), (W.H., N.S., P.K.J., M.M., J.E., R.H., S.L., C.A.R., M.J.L., C.B.) and Medical Research Council-Population Health Research Unit (MRC-PHRU) (P.K.J., J.E., R.H., S.L., C.B.), Nuffield Department of Population Health (NDPH), University of Oxford, United Kingdom; Centre for Nephrology, University College London, United Kingdom (D.C.W.); Dunedin School of Medicine, University of Otago, New Zealand (R.W.); Newcastle-upon-Tyne Hospitals NHS Foundation Trust, United Kingdom (C.T.); National Institute of Diabetes and Digestive and Kidney Diseases, National Institutes of Health, Bethesda, MD (L.A.); and Department of Nephrology, Transplantation and Internal Medicine, Medical University of Silesia, Katowice, Poland (A.W.).

**Keywords:** blood pressure, chronic kidney disease, epidemiology, vascular disease, troponin

## Abstract

Among those with moderate-to-advanced chronic kidney disease, the relationship between blood pressure (BP) and cardiovascular disease seems U shaped but is loglinear in apparently healthy adults. The SHARP (Study of Heart and Renal Protection) randomized 9270 patients with chronic kidney disease to ezetimibe/simvastatin versus matching placebo and measured BP at each follow-up visit. Cox regression was used to assess the association between BP and risk of cardiovascular disease among (1) those with a self-reported history of cardiovascular disease and (2) those with no such history and, based on plasma troponin-I concentration, a low probability of subclinical cardiac disease. A total of 8666 participants had a valid baseline BP and troponin-I measurement, and 2188 had at least 1 cardiovascular event during follow-up. After adjustment for relevant confounders, the association between systolic BP and cardiovascular events was U shaped, but among participants without evidence of previous cardiovascular disease, there was a positive loglinear association throughout the range of values studied. Among those with the lowest probability of subclinical cardiac disease, each 10 mm Hg higher systolic BP corresponded to a 27% increased risk of cardiovascular disease (hazard ratio, 1.27; 95% confidence interval, 1.11–1.44). In contrast, the relationship between diastolic BP and cardiovascular risk remained U shaped irrespective of cardiovascular disease history or risk of subclinical disease. In conclusion, the lack of a clear association between systolic BP and cardiovascular risk in this population seems attributable to confounding, suggesting that more intensive systolic BP reduction may be beneficial in such patients.

**Clinical Trial Registration—:**

URL: http://www.clinicaltrials.gov. Unique identifier: NCT00125593.

## Abstract 2

Supplemental Digital Content is available in the text.

In apparently healthy adults, each 20 mm Hg increase in long-term average—usual—systolic blood pressure (SBP) or 10 mm Hg higher usual diastolic blood pressure (DBP) is associated with about a doubling in the risk of death from ischemic heart disease, stroke, or heart failure, with no threshold level below which lower SBP is not associated with lower risk (at least down to 115/75 mm Hg).^1^ Meta-analyses of randomized trials have demonstrated that lowering SBP reduces cardiovascular risk, confirming that the relationship between blood pressure (BP) and cardiovascular risk is one of the cause and effect.^2,3^

Chronic kidney disease (CKD) is a cause of hypertension and is associated with a high risk of cardiovascular disease.^4^ Most patients with CKD die before reaching end-stage renal disease, and cardiovascular disease is the single largest cause of death among such patients.^4^ However, in contrast to studies in apparently healthy people, observational studies of people with CKD have not consistently yielded a positive association between BP and cardiovascular risk, and at low–normal BP, some studies have indicated an increased risk of cardiovascular disease.^5–10^ It has been suggested that this observation may be attributable to reverse causality, whereby long-standing hypertension causes changes in cardiac structure and function which lower BP while also increasing cardiovascular risk.^11,12^

If such a mechanism is indeed responsible, then it may be hypothesized that a positive association between BP and cardiovascular disease might be present among selected patients with CKD but without cardiac disease. Among patients with advanced CKD (ie, stages 4–5), at least 50% have echocardiographic evidence of abnormal cardiac structure,^13,14^ many without any obvious clinical manifestations.^15^ A potential surrogate measure of subclinical cardiac disease is provided by plasma troponin concentration, which correlates positively with left ventricular mass,^16,17^ correlates negatively with cardiac function,^18^ and predicts development of heart failure in unselected populations^19,20^ and in people with CKD.^21^ We hypothesized that there would be a trend toward a more strongly positive association between BP and cardiovascular events among those with the lowest baseline troponin-I concentrations (and hence the lowest risk of subclinical cardiac disease) in SHARP (Study of Heart and Renal Protection), a randomized trial comparing the combination of ezetimibe plus simvastatin versus placebo among 9270 patients with CKD.^22^

## Methods

The trial methods and results have been published previously.^22^ Patients aged 40 years or over were eligible to participate if they had at least 2 previous measurements of serum or plasma creatinine ≥150 µmol/L (≥1.7 mg/dL) in men or ≥130 µmol/L (≥1.5 mg/dL) in women or were receiving maintenance dialysis. Individuals with a previous history of myocardial infarction or coronary revascularization were excluded, but individuals with a history of angina, peripheral vascular disease, stroke, or diabetes mellitus were eligible. In the current analyses, baseline information refers to information that was recorded at randomization to ezetimibe/simvastatin versus placebo (or shortly before). Baseline information included sociodemographic characteristics (age, sex, ethnicity, and highest attained educational achievement), anthropometric measurements, self-reported medical history, current medication (including antihypertensive treatments, but not their doses), and lifestyle behaviors (alcohol consumption and smoking).

At each study clinic visit, using a suitably sized cuff attached to an automated digital sphygmomanometer (UA-767; A&D Company, Ltd, Tokyo, Japan), trained research nurses recorded a single BP reading after the patient had been seated for 5 minutes.

Baseline samples of nonfasting blood and urine were collected and stored at or below −40°C before transfer to the accredited central laboratory. Creatinine was measured using a kinetic alkaline picrate method calibrated using material traceable to National Institute of Standards and Technology Standard Reference Material 914a, and estimated glomerular filtration rate (eGFR) calculated using the CKD-EPI study (CKD Epidemiology Collaboration) equation.^23^ Troponin-I was measured by chemiluminescent immunoassay on an ACCESS2 analyzer using AccuTnI reagent and calibrator (Beckman Coulter Inc) and Liquichek Cardiac Markers Plus Controls (Bio-Rad Laboratories Ltd). Assay linearity and functional sensitivity was verified down to at least 0.01 ng/mL.

After randomization, participants were followed up at 2 and 6 months and then at 6 monthly intervals for at least 4 years. Wherever possible, follow-up of patients who were unable to attend clinics was conducted by telephone. At each follow-up, information on all serious adverse events (including all hospitalizations) was sought, and further supporting documentation collected on events that might have represented a study outcome. These documents were sent for central adjudication by trained clinicians blind to randomized treatment allocation using prespecified criteria. For the purpose of the present analyses, we defined the following outcomes (1) atherosclerotic cardiovascular event (myocardial infarction, coronary death, unstable angina, ischemic heart failure, coronary revascularization, nonhemorrhagic stroke, transient ischemic attack, and peripheral arterial disease diagnosis, including noncoronary revascularization), (2) nonatherosclerotic cardiovascular event (other cardiac death, nonischemic heart failure, arrhythmia, valvular heart disease, and hemorrhagic stroke), and (3) any cardiovascular event (atherosclerotic and nonatherosclerotic cardiovascular events combined). Analyses of nonvascular mortality were included for comparison.

### Statistical Analysis

The relationship between baseline troponin-I (≤0.01 ng/mL; >0.01 but ≤0.03 ng/mL; and >0.03 ng/mL) and risk of cardiovascular events in the SHARP trial was assessed in Cox models adjusting for age, sex, ethnicity (white, black, Asian, and other), country, highest attained educational achievement (university, secondary school, vocational qualification, other, and unrecorded), smoking (never, former, and current), self-reported diabetes mellitus, body mass index, renal replacement therapy status (dialysis or not), eGFR, BP, and randomized treatment allocation.

Assumptions about the nature and direction of any causal or effect modifying relationships between baseline characteristics, BP, and outcomes were formulated a priori (see directed acyclic graph in Figure S1 in the online-only Data Supplement).^24^ SBP, DBP, and their difference (pulse pressure [PP]) as continuous variables were related to the risk of cardiovascular events using Cox proportional hazards regression adjusted for previous cardiovascular disease and the same variables used in the troponin model above. Because our a priori assumption was that urinary albumin excretion is a mediating variable (ie, BP influences risk partly through its effects on urinary albumin excretion; Figure S1), we did not adjust for this variable in our primary model, although we did so in exploratory analyses. To adjust for variation in BP, we applied a standard correction for regression dilution bias.^25^ Such adjustment allows the relevance of long-term average—usual—BP to be quantified but does not affect the statistical assessment of nonlinearity (Methods in the online-only Data Supplement; Figure S2).^26^ To test for nonlinear associations, models for the main analyses were additionally fitted with a quadratic BP function. A quadratic function was retained if the difference in twice the log-likelihood statistic between 2 nested models (one with and the other without the quadratic function) provided statistical evidence for improvement in model fit (ie, there was evidence of a nonlinear association), and the *P* value for this comparison referred to as the test for nonlinearity. Heterogeneity testing was performed to assess whether associations differed between participants by the selected subgroups (reported cardiovascular disease versus none; and among those with no such report, by troponin-I ≤0.01 versus >0.01 ng/mL) using an analogous method, including where relevant an additional interaction term between evidence of previous cardiovascular disease and a quadratic function of BP.

In figures displaying associations between BP and risk, for each subgroup, hazard ratios (HRs) were presented for 3 groups containing an equal numbers of events with regression lines calculated from regression models using BP as a continuous variable, and these plotted against the mean BP value at the study midpoint accompanied by a confidence interval (CI) derived only from the variance of the log risk in that 1 group. Hence, each HR, including that for the reference group, was associated with a group-specific CI that reflects the amount of data only in that 1 group, thereby allowing appropriate statistical comparisons to be made between any 2 groups.^27^

Values for the small number of missing eGFR and urinary albumin:creatinine ratio were imputed using multiple imputation, with the results across imputations combined using the methods of Rubin.^28^ In sensitivity analyses, the main analyses were repeated separately among participants on dialysis and those not, and among those above and below the study’s median age. The proportional hazard assumption was tested through examination of the time dependency of the Schoenfeld partial residuals. Analyses used SAS v9.3 (SAS Institute, Cary, NY) and R v2.14.2.

## Results

**Figure 1.**
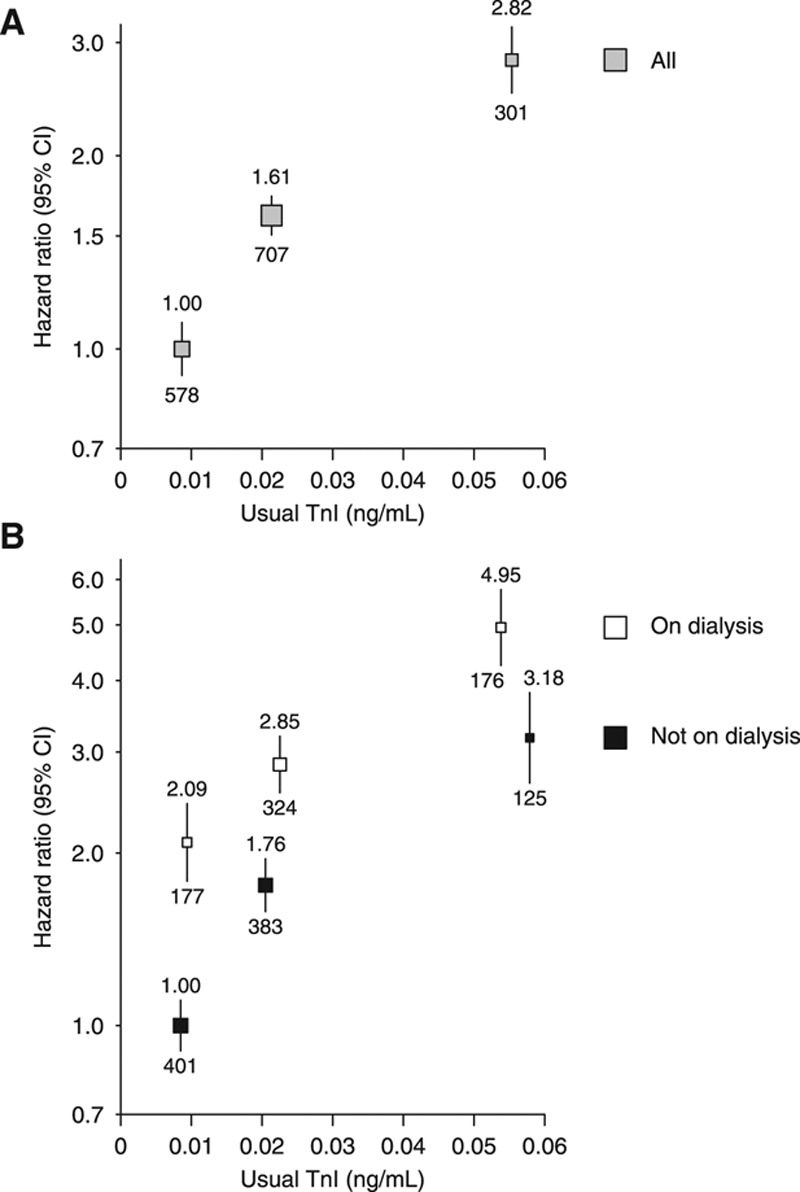
Association between troponin-I (TnI) and risk of cardiovascular events (**A**) overall and (**B**) by renal replacement therapy status. Analyses restricted to those without previous cardiovascular disease at baseline. The reference group in **A** is those with a TnI ≤0.01 ng/mL and in **B**, it is those not on dialysis at baseline with a TnI ≤0.01 ng/mL. Hazard ratios adjusted for age, sex, ethnicity, country, education, smoking status, previous diabetes mellitus, estimated glomerular filtration rate, renal replacement therapy status (**A** only), body mass index, treatment allocation, and blood pressure are quoted (above squares) with number of events (below squares). CI indicates confidence interval.

A total of 604 participants were excluded from analyses due either to a missing baseline measurement of BP (n=25 individuals) or a missing troponin-I measurement (n=579). Of the remaining 8666 participants, 7278 reported no previous history of cardiovascular disease, and among this group, a higher baseline troponin-I was associated with male sex, higher SBP, older age, more diabetes mellitus, and worse renal function (with a larger proportion of such patients on dialysis; Table S1). After adjustment for these differences, increasing baseline troponin-I was strongly associated with future cardiovascular risk. Compared with those with a troponin-I ≤0.01 ng/mL, those with troponin-I concentration >0.01 but ≤0.03 ng/mL, and >0.03 ng/mL were at 61% (HR, 1.61; 95% CI, 1.43–1.81) and 182% (HR, 2.82; 95% CI, 2.42–3.28) increased cardiovascular risk, respectively (Figure 1A). A higher troponin-I was associated with increased cardiovascular risk in both dialysis and nondialysis patients (Figure 1B).

Mean baseline SBP ranged from 116 mm Hg in the lowest third to 163 mm Hg in the highest third. Compared with those in the lowest third, those in the highest third of SBP were more often male, were older, and reported more diabetes mellitus and previous cardiovascular disease, and nondialysis patients had lower eGFR (Table; Table S2). Mean baseline DBP ranged from 65 mm Hg in the lowest third to 93 mm Hg in the highest third. Compared with those in the lowest third of DBP, and in contrast to the baseline characteristics by SBP, those with higher DBP were younger, less likely to report diabetes mellitus, previous cardiovascular disease, or to be on dialysis (Table; Table S2). The majority of participants were taking at least 1 antihypertensive agent, ranging from 87% in the highest third of baseline SBP to 81% in the lowest third, and from 86% to 83% in the highest and lowest thirds of DBP, respectively (Table). Over one half of participants were taking at least 2 agents (Table S2).

**Table 1.**
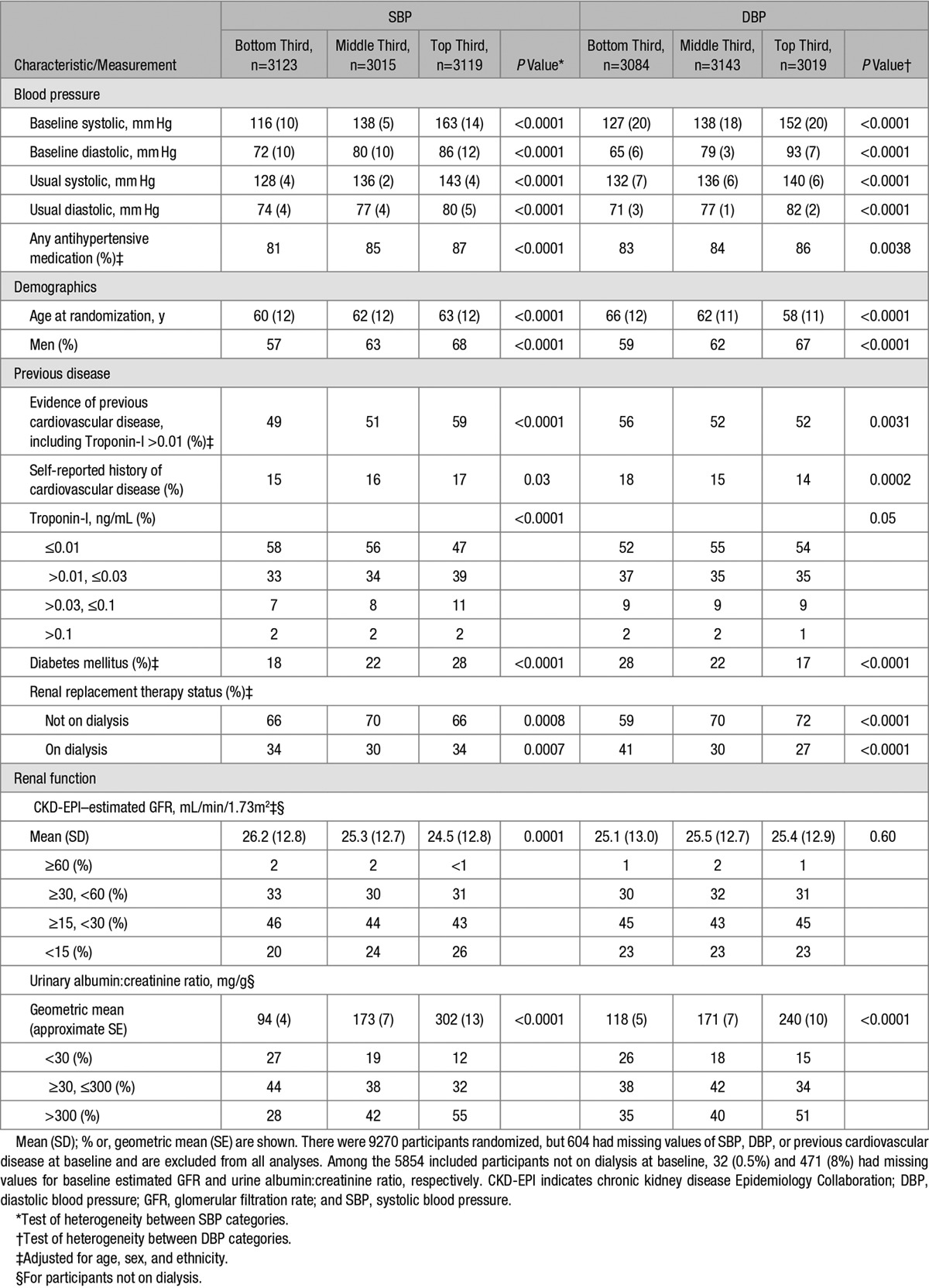
Baseline Characteristics and Measurements by Thirds of Baseline Blood Pressure

Overall, 2188 participants experienced at least 1 cardiovascular event during a median of 4.9 years of follow-up (annual rate 6.7% per year).

### SBP and Vascular Risk

**Figure 2.**
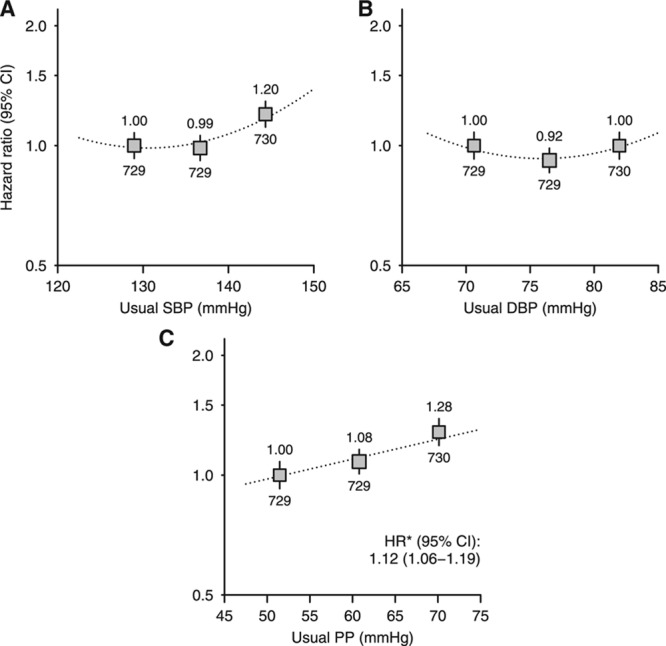
Association between (**A**) systolic blood pressure (SBP), (**B**) diastolic blood pressure (DBP), and (**C**) pulse pressure (PP) and cardiovascular events overall. For each plot, categories of blood pressure contain similar numbers of events. Hazard ratios (HRs) adjusted for age, sex, ethnicity, country, education, smoking status, previous cardiovascular disease, previous diabetes mellitus, estimated glomerular filtration rate, renal replacement therapy status, body mass index, and treatment allocation are quoted (above squares) with numbers of events (below). Exclusions as per Table.*HRs per 10 mm Hg higher usual blood pressure are presented for associations where there is no evidence of deviation from a loglinear relationship. CI indicates confidence interval.

**Figure 3.**
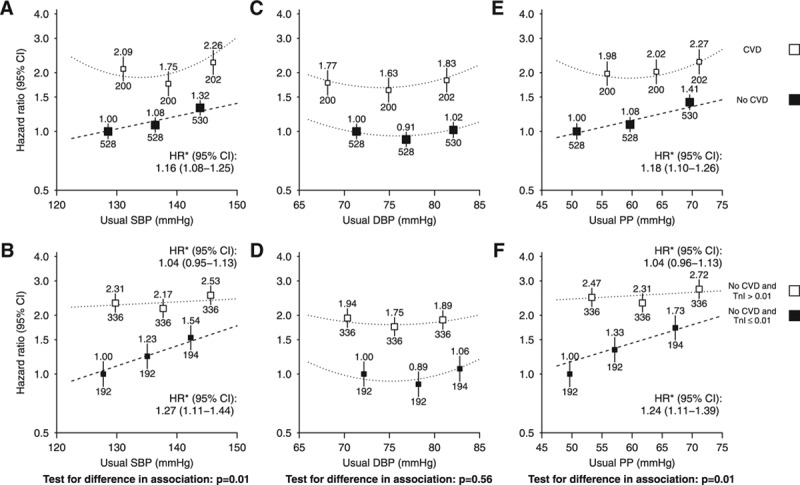
Association between systolic blood pressure (SBP), diastolic blood pressure (DBP), and pulse pressure (PP) and cardiovascular events, subdivided by self-reported history of previous cardiovascular disease (**A**, **C**, **E**) and by baseline troponin-I concentration (**B**, **D**, **F**). For each plot, categories of blood pressure contain similar numbers of events. Hazard ratios adjusted for age, sex, ethnicity, country, education, smoking status, previous diabetes mellitus, estimated glomerular filtration rate, renal replacement therapy status, body mass index, and treatment allocation are quoted (above squares) with numbers of events (below). Exclusions as per Table. *Hazard ratios per 10 mm Hg higher usual SBP/PP are presented for associations where there is no evidence of deviation from a log–linear relationship. CI indicates confidence interval; CVD, self-reported history of cardiovascular disease; HR, hazard ratio; and TnI, troponin-I (ng/mL).

The adjusted association between SBP and cardiovascular risk was U shaped (Figure 2A; test against the linearity assumption [nonlinearity] *P*=0.003). But, among the 7278 participants who reported no previous history of cardiovascular disease, there was a positive loglinear association throughout the range studied (Figure 3A; nonlinearity *P*=0.35). After adjusting for regression dilution, each 10 mm Hg higher usual SBP was associated with 16% higher cardiovascular risk (HR, 1.16; 95% CI, 1.08–1.25). Among this group, there was a steeper association in those with lower baseline troponin (heterogeneity test *P*=0.01; Figure 3B). Among those at lowest probability of cardiac disease (no self-reported previous cardiovascular disease and troponin-I ≤0.01 ng/mL), each 10 mm Hg higher usual SBP was associated with 27% higher cardiovascular risk (HR, 1.27; 95% CI, 1.11–1.44; Figure 3B). Additional adjustment for baseline urinary albumin:creatinine ratio had little impact on this estimated HR (1.23; 95% CI, 1.08–1.40).

**Figure 4.**
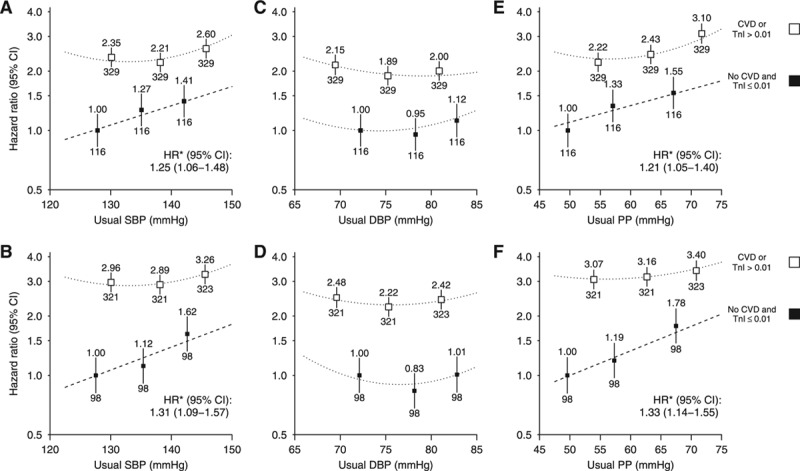
Association between (**A**) systolic blood pressure (SBP), (**C**) diastolic blood pressure (DBP), and (**E**) pulse pressure (PP) and atherosclerotic cardiovascular events and association between (**B**) SBP, (**D**) DBP, and (**F**) PP and nonatherosclerotic cardiovascular events, subdivided by evidence of previous cardiovascular disease. Conventions as per Figure 3. CI indicates confidence interval; CVD, self-reported history of cardiovascular disease; HR, hazard ratio; and TnI, troponin-I (ng/mL).

**Figure 5.**
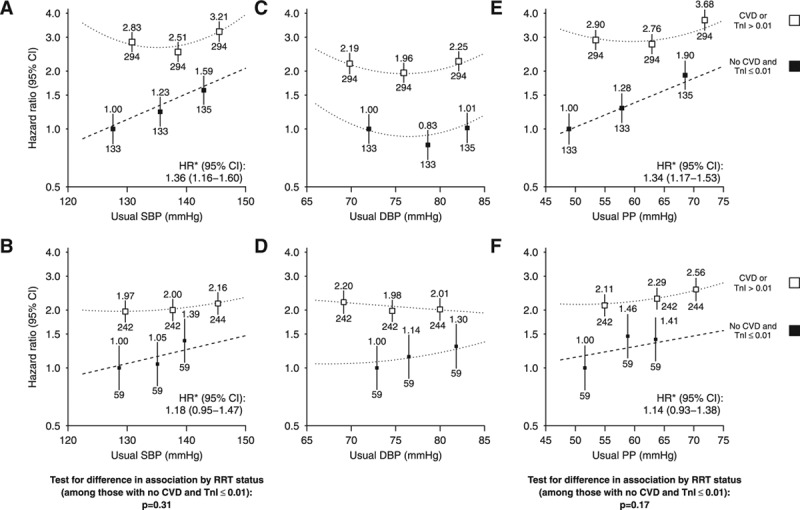
Association between systolic blood pressure (SBP), diastolic blood pressure (DBP), and pulse pressure (PP) and cardiovascular events, subdivided by evidence of previous cardiovascular disease, for those not on dialysis (**A**, **C**, **E**) and on dialysis (**B**, **D**, **F**). Conventions as per Figure 3. CI indicates confidence interval; CVD, self-reported history of cardiovascular disease; HR, hazard ratio; and TnI, troponin-I (ng/mL).

The magnitude of association between SBP and risk of cardiovascular events was similar for atherosclerotic (HR per 10 mm Hg usual SBP, 1.25; 95% CI, 1.06–1.48) and nonatherosclerotic events (HR per 10 mm Hg usual SBP, 1.31; 95% CI, 1.09–1.57; Figure 4A and 4B). Within the low cardiac risk group, there were apparently similar loglinear associations between SBP and risk of cardiovascular events among those on dialysis and those not (HRs per 10 mm Hg higher SBP 1.36; 95% CI, 1.16–1.60 and 1.18; 95% CI, 0.95–1.47; heterogeneity *P*=0.31; Figure 5A and 5B), although these analyses were constrained by the small numbers of events. Likewise, there were apparently similar loglinear associations in those younger than 62 and those aged 62 years or over (HRs, 1.37; 95% CI, 1.14–1.66; 1.20; 95% CI, 1.00–1.43; heterogeneity *P*=0.31; Figure S3A and S3B).

### DBP and Vascular Risk

Overall, there was a U-shaped association between DBP and cardiovascular events (nonlinearity *P*=0.0008; Figure 2B). This association was U shaped irrespective of a recorded history of cardiovascular disease or the probability of cardiac disease in those without such a history (Figure 3C and 3D) and was similar for both atherosclerotic and nonatherosclerotic events (Figure 4C and 4D), in dialysis and nondialysis (Figure 5C and 5D), and in younger and older patients (Figure S3C and S3D).

### PP and Vascular Risk

Overall, the adjusted association between PP and risk of cardiovascular events was loglinear (HR per 10 mm Hg higher usual PP, 1.12; 95% CI, 1.06–1.19; Figure 2C) but was U shaped among those with a history of cardiovascular disease and loglinear among those without such a history (HR per 10 mm Hg higher usual PP, 1.18; 95% CI, 1.10–1.26; Figure 3E). Among those in the lowest category of troponin-I, each 10 mm Hg higher usual PP was associated with 24% higher cardiovascular risk (HR, 1.24; 95% CI, 1.11–1.39; Figure 3F), with similar relationships for atherosclerotic and nonatherosclerotic cardiovascular events considered separately (HRs per 10 mm Hg higher usual PP 1.21; 95% CI, 1.05–1.40 and 1.33; 95% CI, 1.14–1.55, respectively; Figure 4E and 4F). Among those at lowest cardiac risk, the HRs per 10 mm Hg higher PP were similar among dialysis and nondialysis (Figure 5E and 5F) and in younger and older patients (Figure S3E and S3F).

### BP and Nonvascular Mortality

There were 1196 nonvascular deaths during follow-up (3.2% per year). For SBP, there was some evidence for a U-shaped association (nonlinearity *P*=0.03) with nonvascular mortality, while the relationship with DBP appeared flat (nonlinearity *P*=0.24; HR per 5 mm Hg usual DBP, 1.00; 95% CI, 0.94–1.06) and was similar irrespective of baseline troponin-I (Figure S4).

## Discussion

A U-shaped association between BP and cardiovascular risk has been observed in many studies of populations with advanced CKD,^5–10^ which is in contrast to the positive loglinear relationships with ischemic heart disease, stroke, and heart failure mortality observed among apparently healthy adults.^1^ The presence of a clear positive loglinear relationship between SBP (or PP) and cardiovascular events in patients with CKD at lowest risk of cardiac disease in SHARP suggests that reverse causality is a plausible explanation for previously observed U-shaped associations among patients with moderate-to-advanced CKD.^5–10^ A loglinear relationship between SBP (or PP) and the risk of cardiovascular events was present in both dialysis and nondialysis patients, suggesting that BP remains a cause of cardiovascular disease irrespective of the severity of CKD, and hence that the absolute benefits of lowering BP among dialysis patients may be larger than those achievable at an earlier stage of CKD.

We did not observe a positive association between DBP and cardiovascular risk in this population. Myocardial perfusion is dependent on diastolic blood flow, and it has been suggested that a hypertrophied left ventricle (a key feature of structural heart disease in CKD^13,14^) may be more likely to become ischemic at low levels of DBP than a normal ventricle.^29^ Because PP is the difference between SBP and DBP, our finding of a positive association between PP and cardiovascular risk in those at lowest risk of cardiac disease reflects the finding of a positive relationship for SBP and a U-shaped relationship for DBP. Vascular calcification is accelerated in CKD and reduces vascular recoil, thereby increasing SBP and decreasing DBP, that is, widening PP.^30^ If present, vascular calcification may increase the risk of cardiovascular events,^31^ and the present analyses suggest that widening PP is associated with an increased risk of both atherosclerotic and nonatherosclerotic cardiovascular events in this population.

Among people with cardiovascular disease, randomized trials have shown that lowering BP is effective at reducing cardiovascular risk,^32^ in spite of U-shaped associations between BP and cardiovascular risk being commonly observed in such populations.^29,33–35^ Similarly, lowering BP is effective in elderly people,^36,37^ in whom some prospective studies have also failed to demonstrate a positive association between BP and cardiovascular disease.^29^ Comparatively few people with moderate-to-advanced CKD have been studied in trials of antihypertensive therapy, but about 10 000 people with some evidence of reduced renal function were included in a recent meta-analysis.^3^ In this study, each 5 mm Hg SBP reduction lowered cardiovascular risk by 14%, with no heterogeneity in this risk reduction among different categories of eGFR.^3^ Similar benefits were observed in a separate meta-analysis of trials conducted among people on dialysis.^38^ However, although BP lowering seems beneficial in CKD, the optimum BP target for people with CKD is unknown, with current guideline recommendations ranging from <130/80 to <150/90 mm Hg (Table S3).

There have been 2 negative trials of intensive versus standard BP lowering in CKD populations, but these lacked statistical power to detect the magnitude of benefit suggested by our analyses.^39,40^ The recent SPRINT (Systolic Blood Pressure Intervention Trial) demonstrated clearly that an SBP target of 120 mm Hg (achieved SBP 121 mm Hg) was superior to a target of 140 mm Hg (achieved SBP 136 mm Hg) in high-risk adults.^37^ These data, taken together with the evidence of reverse causality in the present analysis in the SHARP trial, suggest that trials of lower BP targets in patients with CKD are indicated. Such trials would also be able to assess the potential hazards of lower BP targets—for example, in SPRINT, the more intensive BP regimen was associated with an excess of acute kidney injury (204/4678 [4.4%] versus 120/4683 [2.6%]; *P*<0.001)^37^—and the somewhat uncertain benefits of intensive BP lowering on renal progression.

Our study has the advantage of a large sample size, detailed adjudication of cardiovascular events, and the ability to select those at lowest risk of cardiac disease through the measurement of baseline troponin (which has not been possible in previous studies^5–10^). The most important limitation is that, because no cardiac imaging was performed in SHARP, the correlation between troponin-I concentration and preexisting structural cardiac disease cannot be formally confirmed in this cohort. Nevertheless, the use of troponin as a tool to identify those at higher risk of subclinical cardiac disease is supported by other studies,^16–21,41^ and baseline troponin-I was a strong independent predictor of cardiovascular risk in both dialysis and nondialysis patients in SHARP. A further limitation is that SHARP only had a single measurement of BP at each clinic visit, which means short-term variability in BP was not assessed. This may also lead to underestimates of the strength of the relationship between BP and cardiovascular risk, particularly because BP exhibits marked day-to-day variability among people on dialysis in whom out-of-dialysis unit SBP readings give better estimates of average BP than measurements taken before or after dialysis.^42,43^ This limitation was partially offset by our adjustment for regression dilution bias. Such adjustment is well established in studies of apparently healthy individuals^25^ because the magnitude of reductions in cardiovascular risk produced by antihypertensive therapy in randomized trials^2,3^ is better predicted by associations between usual, rather than a single measure of BP in observational studies.^1–3^

## Perspectives

In summary, a U-shaped association between SBP and cardiovascular risk in CKD populations, as observed in many previous studies, may be attributable to reverse causality because of subclinical cardiac disease. When adjustment is made for such confounding, the observed association between SBP and both atherosclerotic and nonatherosclerotic cardiovascular risk is positive and loglinear, consistent with BP being a causal risk factor for both forms of cardiovascular disease in patients with CKD, as it is in other populations. Randomized trials of more intensive BP reduction should be a priority in patients with moderate-to-advanced CKD.

## Acknowledgments

We thank the participants in SHARP trial (Study of Heart and Renal Protection) and the local clinical center staff, regional and national coordinators, steering committee (Materials), and data monitoring committee.

## Sources of Funding

The SHARP study (Study of Heart and Renal Protection) was funded by Merck & Co, Inc, Kenilworth, NJ, with additional support from the Australian National Health Medical Research Council, the British Heart Foundation, and the UK Medical Research Council (which supported this analysis). The funders had no involvement in data collection, analysis, interpretation, article writing, or the decision to submit for publication.

## Disclosures

The Clinical Trial Service Unit and Epidemiological Studies Unit, which is part of the University of Oxford, has a staff policy of not accepting honoraria or consultancy fees.

## Supplementary Material

## Novelty and Significance

### What Is New?

- Observational studies have found that the association between systolic blood pressure (SBP) and cardiovascular risk in chronic kidney disease (CKD) populations is U shaped. We have shown that the U-shaped relationship is confined to patients with a known history of cardiovascular disease or a high probability of such disease, whereas among patients with a lower probability of subclinical cardiovascular disease, there is a loglinear association between SBP (or pulse pressure) and both atherosclerotic and nonatherosclerotic cardiovascular diseases.

### What Is Relevant?

- These observations indicate that confounding by disease is the chief explanation for the apparent weakening and reversal of the association between SBP and cardiovascular risk in moderate-to-advanced CKD and suggest that such confounding masks a causal association between blood pressure and risk in patients with CKD with established cardiovascular disease. They support the need for randomized trials of more versus less intensive blood pressure reduction among patients with moderate-to-advanced CKD, including hemodialysis and peritoneal dialysis patients.

### Summary

This study examined the association between blood pressure and risk of cardiovascular disease among CKD patients with (1) no self-reported history of cardiovascular disease and (2) no such history and, based on plasma troponin-I concentration, a low probability of subclinical cardiac disease. Overall, the association between SBP and cardiovascular events was U shaped, but among participants without evidence of previous cardiovascular disease, there was a positive loglinear association with SBP throughout the range of values studied. Among those with the lowest probability of subclinical cardiac disease, each 10 mm Hg higher SBP corresponded to a 27% increased risk of cardiovascular disease.
