# Supplementary material for: Evidence for Reverse Causality in the Association Between Blood Pressure and Cardiovascular Risk in Patients With Chronic Kidney Disease
Source: Hypertension. 2017 Jan 11;69(2):314–22. doi: 10.1161/HYPERTENSIONAHA.116.08386 (PMC5222554; doi:10.1161/HYPERTENSIONAHA.116.08386)
Supplement: Supplementary file 1 [file hyp-69-314-s001.pdf]

Evidence for reverse causality in the association between blood pressure and cardiovascular risk in patients with chronic kidney disease

William Herrington[1]* MD, Natalie Staplin[1]* PhD, Parminder K Judge[1,2] MBChB, Marion Mafham[1] MD, Jonathan Emberson[1,2] PhD, Richard Haynes[1,2] DM, David C Wheeler[3] FRCP, Robert Walker[4] FRACP, Charlie Tomson[5] DM, Larry Agodoa[6] FACP, Andrzej Wiecek[7] FRCP, Sarah Lewington[1,2] DPhil, Christina A Reith[1] FRCP, Martin J Landray[1] FRCP and Colin Baigent[1,2] FRCP, on behalf of the SHARP Collaborative Group. *Joint contribution

1. Clinical Trial Service Unit and Epidemiological Studies Unit (CTSU),
Nuffield Department of Population Health (NDPH), University of Oxford, UK
2. Medical Research Council-Population Health Research Unit (MRC-PHRU),
NDPH, University of Oxford, UK
3. Centre for Nephrology, University College London, London, UK
4. Dunedin School of Medicine, University of Otago, Otago, New Zealand
5. Newcastle-upon-Tyne Hospitals NHS Foundation Trust, Newcastle, UK
6. National Institute of Diabetes and Digestive and Kidney Diseases,
National Institutes of Health, Bethesda, MD, USA
7.Department of Nephrology, Transplantation and Internal Medicine,
Medical University of Silesia, Katowice, Poland

Prof. Colin Baigent
MRC-PHRU, NDPH, Richard Doll Building, Old Road Campus,
Roosevelt Drive, Oxford OX3 7LF, UK
Email:colin.baigent@ndph.ox.ac.uk
Phone: +44-1865-743743; Fax:+44-1865-743985

Detailed Statistical Methods: Estimation of "Usual" Blood Pressure

To ensure natural blood pressure variation and any measurement error was accounted for, a standard correction for such regression-dilution bias was made. (Supplemental Figure S2).[1,2] Each individual's usual systolic blood pressure, S, was estimated using linear regression models with blood pressure at the study midpoint (2.5 years) as the outcome and their baseline value, s, as the explanatory variable. It was found that there was a quadratic relationship between baseline and follow-up blood pressure, so usual systolic blood pressure was estimated using the formula:

$$S=136.1 + 0.316(s - 138.9) - 0.001(s - 138.9)^2.$$

Similarly, each individual's usual diastolic blood pressure, D, was calculated from their baseline value, d, using the formula:

$$D=77.1 + 0.396(d - 79.1) - 0.0018(d - 79.1)^2.$$

A similar method of estimation of usual blood pressure has been used previously in the analyses of the influence of blood pressure on vascular disease risk performed by the Prospective Studies Collaboration.[3]

The following hazard ratios demonstrate how the use of a single blood pressure measurement or the average of 3 readings over 6 months would underestimate the relevance of SBP to vascular risk (among those who reported no prior history of cardiovascular disease and a baseline troponin-I ≤0.01ng/mL) compared to using the usual SBP described above.

	**Hazard ratio (95% CI) per 10 mmHg higher SBP**
"Usual" SBP	1.29 (1.12-1.48)*
Average SBP of 3 readings over 6 months	1.11 (1.05-1.16)
Single baseline measure of SBP	1.08 (1.04-1.13)

SBP = systolic blood pressure. *The hazard ratio quoted here for "usual" SBP differs to that quoted in Figure 3 as these analyses exclude participants with missing values of SBP at 2 or 6 months.

Statistical References

1. Clarke R, Shipley M, Lewington S, Youngman L, Collins R, Marmot M, Peto R. Underestimation of risk associations due to regression dilution in long-term follow-up of prospective studies. *Am J Epidemiol*. 1999;150:341-353.
2. Carroll RJ RD, Stefanski LJ, Crainiceanu CM *Measurement error in nonlinear models: A modern perspective*. Boca Raton: CRC Press.; 2006.
3. Prospective Studies Collaboration. Age-specific relevance of usual blood pressure to vascular mortality: a meta-analysis of individual data for one million adults in 61 prospective studies. *Lancet*, 2002; 360:1903-1913.

Table S1: Baseline characteristics and laboratory measurements subdivided by self-reported history of prior cardiovascular disease and baseline troponin-I concentration

	Self-reported history of prior cardiovascular disease and baseline troponin-I concentration		
	No CVD		
**Characteristic/measurement**	**TnI≤0.01 (n=4070)**	**TnI>0.01 (n=3208)**	**CVD (n=1388)**
**Blood pressure**			
Baseline systolic (mmHg)	136 (20)	142 (23)	141 (23)
Baseline diastolic (mmHg)	80 (12)	79 (13)	76 (13)
Usual systolic (mmHg)	135 (6)	136 (7)	136 (7)
Usual diastolic (mmHg)	77 (5)	77 (5)	76 (5)
Any antihypertensive medication	3405 (84%)	2722 (85%)	1194 (86%)
**Demographics**			
Age at randomization (years)	59 (11)	64 (12)	67 (11)
Men	2338 (57%)	2193 (68%)	910 (66%)
Ethnicity			
White	2985 (73%)	2222 (69%)	1033 (74%)
Black	73 (2%)	104 (3%)	41 (3%)
Asian	908 (22%)	786 (25%)	272 (20%)
Other	104 (3%)	96 (3%)	42 (3%)
Education			
University	569 (14%)	307 (10%)	120 (9%)
Secondary school	1377 (34%)	1016 (32%)	435 (31%)
Vocational qualifications	891 (22%)	768 (24%)	366 (26%)
Primary school or no formal education	651 (16%)	647 (20%)	287 (21%)
Not specified	582 (14%)	470 (15%)	180 (13%)
Current smoker	560 (14%)	387 (12%)	207 (15%)
**Prior disease**			
Self reported history of vascular disease	0 (0%)	0 (0%)	1388 (100%)
Troponin-I (ng/mL)			
≤0.01	4070 (100%)	0 (0%)	527 (38%)
>0.01 to ≤0.03	0 (0%)	2502 (78%)	551 (40%)
>0.03 to ≤0.1	0 (0%)	591 (18%)	186 (13%)
>0.1	0 (0%)	115 (4%)	40 (3%)
Diabetes	621 (15%)	859 (27%)	506 (36%)
Renal status			
Not on dialysis	3187 (78%)	1731 (54%)	926 (67%)
On dialysis	878 (22%)	1474 (46%)	460 (33%)
**Measurements**			
CKD-EPI-estimated GFR (mL/min/1.73m²)*			
Mean (SD)	26.6 (13.3)	23.2 (12.0)	25.0 (13.0)
≥60	49 (1%)	20 (1%)	13 (1%)
≥30 to <60	1127 (28%)	426 (13%)	263 (19%)
≥15 to <30	1375 (34%)	790 (25%)	411 (30%)
<15	639 (16%)	498 (16%)	211 (15%)
Urinary albumin:creatinine ratio (mg/g)*			
Median (IQR)	175 (37-645)	253 (60-896)	224 (49-979)
<30	648 (16%)	267 (8%)	153 (11%)
≥30 to ≤300	1159 (28%)	596 (19%)	293 (21%)
>300	1152 (28%)	752 (23%)	363 (26%)
Body-mass index (kg/m²)	27.0 (5.3)	27.0 (5.7)	27.4 (5.6)
**Treatment allocation**			
Randomized to simvastatin plus ezetimibe	2014 (49%)	1630 (51%)	709 (51%)

Mean (SD) or n (%) shown. GFR=glomerular filtration rate. CVD = self-reported history of cardiovascular disease. TnI=troponin-I.
*For participants not on dialysis. Missing data as described in Table 1.

Table S2: Additional baseline characteristics and laboratory measurements by tertiles of baseline blood pressure

	Systolic blood pressure (SBP)				Diastolic blood pressure (DBP)			
Characteristic/measurement	Bottom third (n=3123)	Middle third (n=3015)	Top third (n=3119)	P value*	Bottom third (n=3084)	Middle third (n=3143)	Top third (n=3019)	P value†
**Other demographics**								
Ethnicity				<0.0001				0.0016
White	74%	74%	67%		74%	72%	69%	
Black	3%	2%	3%		3%	3%	3%	
Asian	20%	21%	26%		21%	23%	24%	
Other	3%	3%	3%		3%	3%	4%	
Education				<0.0001				0.21
University	13%	12%	10%		11%	12%	12%	
Secondary school	32%	34%	31%		32%	34%	33%	
Vocational qualifications	22%	23%	24%		24%	22%	23%	
Primary school or no formal education	17%	17%	21%		19%	18%	19%	
Not specified	15%	14%	14%		15%	14%	14%	
Current smoker	12%	13%	14%	0.07	13%	12%	14%	0.03
**Medications**								
Number of antihypertensive medications				<0.0001				0.01
None	19%	15%	13%		17%	17%	14%	
One	26%	23%	23%		23%	25%	24%	
Two	24%	26%	25%		23%	25%	28%	
Three or more	30%	36%	38%		38%	33%	34%	
Type of antihypertensive medication								
ACE inhibitor or ARB	53%	55%	55%	0.12	54%	54%	55%	0.42
Beta blocker	36%	38%	39%	0.03	38%	36%	39%	0.03
Calcium channel blocker	32%	43%	48%	<0.0001	40%	41%	43%	0.09
Diuretic	41%	41%	42%	0.50	45%	40%	39%	<0.0001
Other co-medication								
Antiplatelet therapy	23%	22%	23%	0.88	27%	21%	19%	<0.0001
Oral anticoagulant therapy	4%	3%	3%	0.0021	4%	3%	3%	0.03
Erythropoiesis stimulating agent	26%	26%	29%	0.01	31%	26%	25%	<0.0001
Sevelamer	9%	7%	8%	0.17	10%	7%	7%	<0.0001

Table S2: Additional baseline characteristics and laboratory measurements by tertiles of baseline blood pressure

Characteristic/measurement	Systolic blood pressure (SBP)				Diastolic blood pressure (DBP)			
	Bottom third (n=3123)	Middle third (n=3015)	Top third (n=3119)	P value*	Bottom third (n=3084)	Middle third (n=3143)	Top third (n=3019)	P value†
**Other measurements**								
Body-mass index (kg/m²)	26.8 (5.4)	27.0 (5.4)	27.4 (5.4)	0.0005	27.1 (5.5)	27.2 (5.4)	27.0 (5.5)	0.44
Total cholesterol (mmol/L)	4.83 (1.15)	4.91 (1.15)	4.91 (1.16)	0.01	4.75 (1.17)	4.89 (1.14)	5.02 (1.16)	<0.0001
LDL cholesterol (mmol/L)	2.74 (0.86)	2.80 (0.86)	2.78 (0.86)	0.02	2.68 (0.87)	2.78 (0.85)	2.86 (0.87)	<0.0001
HDL cholesterol (mmol/L)	1.11 (0.33)	1.12 (0.33)	1.13 (0.33)	0.09	1.08 (0.33)	1.12 (0.33)	1.15 (0.33)	<0.0001
Triglycerides (mmol/L)	2.31 (1.73)	2.34 (1.72)	2.32 (1.73)	0.81	2.38 (1.75)	2.28 (1.72)	2.31 (1.75)	0.07
Phosphate (mmol/L)	1.26 (0.44)	1.27 (0.44)	1.30 (0.44)	0.0008	1.30 (0.44)	1.26 (0.44)	1.27 (0.44)	0.0015
Hemoglobin (g/dL)	12.28 (1.66)	12.17 (1.65)	12.05 (1.64)	<0.0001	11.90 (1.66)	12.24 (1.63)	12.37 (1.67)	<0.0001
Albumin (g/L)	40.1 (3.7)	40.2 (3.7)	40.0 (3.7)	0.04	39.9 (3.8)	40.2 (3.7)	40.1 (3.8)	0.0028
C-reactive protein (mg/L) [geometric mean (approximate SE)]	3.1 (0.1)	2.9 (0.1)	3.1 (0.1)	0.09	3.3 (0.1)	2.9 (0.1)	3.0 (0.1)	0.0004
**Treatment allocation**								
Randomized to simvastatin plus ezetimibe	50%	51%	50%	0.52	50%	50%	50%	0.91

Mean (SD) or % shown, all characteristics adjusted for age, sex and ethnicity, with the exception of ethnicity. ACE=angiotensin-converting enzyme. ARB=angiotensin-II receptor blocker. LDL=low-density lipoprotein. HDL=high-density lipoprotein. *P value for test of heterogeneity between SBP categories. †P value for test of heterogeneity beween DBP categories.

Table S3: Guideline recommendations for management of blood pressure in chronic kidney disease

Guideline	Blood pressure target, mmHg	Target population and recommendation
Kidney Disease Improving Global Outcomes (KDIGO, 2012)[1]	≤140/90	CKD[*] or diabetes without microalbuminuria[†]
	≤130/80	CKD[*] or diabetes with micro[†]- or macroalbuminuria[‡]
Eighth Joint National Committee (JNC-8, 2014)[2]	<140/90	18-69 years and eGFR or mGFR <60 mL/min/1.73m^2 with albuminuria[§]
	<150/90	eGFR <60 mL/min/1.73m^2 and ≥70 years[‖], or CKD without albuminuria
European Society of Hypertension (ESH) and the European Society of Cardiology (ESH-ESC, 2013)[3]	<140/90	CKD[¶]
	<130/90	Overt proteinuria[#]
National Institute for Health and Clinical Excellence (NICE, 2014)[4]	<140/90	Non-diabetic CKD[*] without albuminuria[**]
	<130/80	CKD[*] with albuminuria[††]
		CKD[*] with diabetes
American College of Cardiology Foundation and the American Heart Association (ACCF/AHA, 2011)[5]	<130/80	CKD[‡‡] in elderly patients with hypertension
Canadian hypertension education program (CHEP, 2015)[6]	<140/90	Non-diabetic CKD[‡‡]
	<130/80	Diabetic CKD[‡‡]

CKD = chronic kidney disease; eGFR = estimated glomerular filtration rate; mGFR = measured glomerular filtration rate. [*] CKD defined using the National Kidney Foundation Kidney Disease Outcomes Quality Initiative (NFK KDOQI) definition as; either kidney damage (defined as pathological abnormalities or markers of damage, including abnormalities in blood or urine tests or imaging studies) or GFR <60 mL/min/1.73m^2 for ≥3 months; [†] Microalbuminuria defined as urine albumin excretion ≥30-300 mg/d; [‡] Macroalbuminuria defined as urine albumin excretion >300 mg/d; [§] Albuminuria defined as >30 mg/g at any age and at any level of GFR; [‖] If ≥70 years, treatment should be individualised, taking into consideration factors such as frailty, comorbidities and albuminuria; [¶] CKD includes those with reduced renal function and/or the detection of elevated urinary excretion of albumin, staged according to eGFR; [#] Overt proteinuria defined as >300 mg/d; [**] Albuminuria defined as albumin:creatinine ratio ≥30 mg/mmol; [††] Albuminuria defined as albumin:creatinine ratio ≥70 mg/mmol; [‡‡] CKD defined as eGFR <60 mL/min/1.73 m^2

References for Table S3

1. Kidney Disease Improving Global Outcomes (KDIGO). KDIGO Clinical Practice Guideline for the Management of Blood Pressure in Chronic Kidney Disease. *Kidney Int.*2012;2(5).
2. James PA, Oparil S, Carter BL, et al. 2014 Evidence-Based Guideline for the Management of High Blood Pressure in Adults: Report From the Panel Members Appointed to the Eighth Joint National Committee (JNC 8). *JAMA.* 2014;311:507-520.
3. Mancia G, Fagard R, Narkiewicz K, et al. 2013 ESH/ESC Guidelines for the management of arterial hypertension: The Task Force for the management of arterial hypertension of the European Society of Hypertension (ESH) and of the European Society of Cardiology (ESC). *J hypertens*. 2013;31:1281-1357.
4. National Institute for Health and Clinical Excellence (NICE): Guidance. Chronic kidney disease (partial update): Early identification and management of chronic kidney disease in adults in primary and secondary care. Clinical Guideline 182. London: National Institute for Health and Care Excellence (UK) Copyright (c) National Clinical Guideline Centre. 2014. https://www.nice.org.uk/guidance/cg182 (accessed 20th July 2015).
5. Aronow WS, Fleg JL, Pepine CJ, et al. ACCF/AHA 2011 Expert Consensus Document on Hypertension in the Elderly: A report of the American College of Cardiology Foundation Task Force on Clinical Expert Consensus Documents. *J Am Coll Cardiol*. 2011;57:2037-2114.
6. Daskalopoulou SS, Rabi DM, Zarnke KB, et al. The 2015 Canadian Hypertension Education Program Recommendations for Blood Pressure Measurement, Diagnosis, Assessment of Risk, Prevention, and Treatment of Hypertension. *Canadian J Cardiol*. 2015;31:549-568.

Figure S1: Causal diagram showing the assumed associations between baseline blood pressure, outcomes and other characteristics

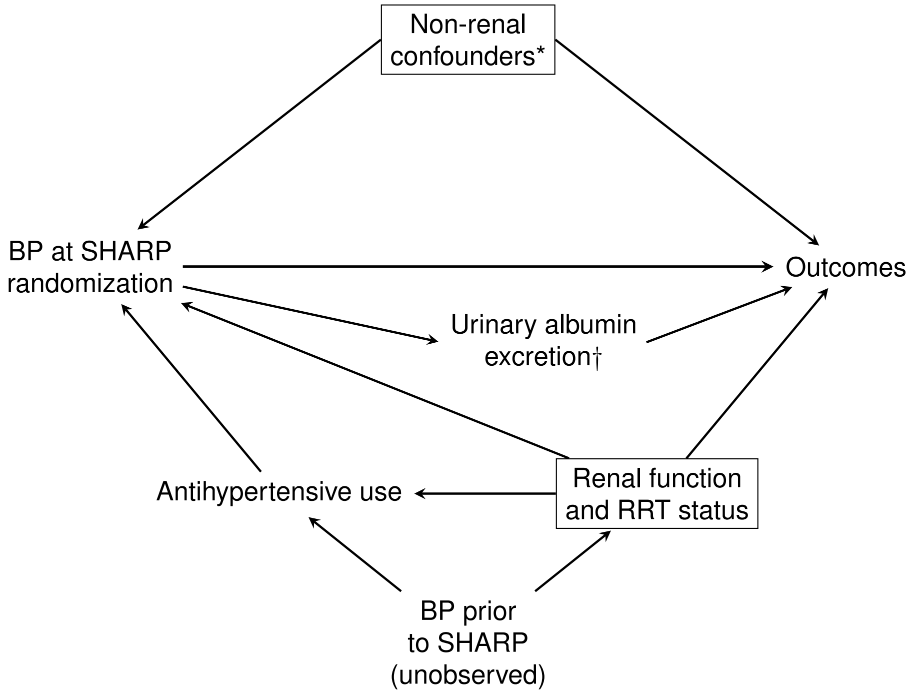

RRT=renal replacement therapy. *Age, sex, ethnicity, country, education, smoking status at screening, prior vascular disease, prior diabetes and body-mass index.

Analyses were adjusted for the confounders enclosed by boxes in the causal diagram. No adjustment was made for antihypertensive use as it was assumed that any effect on outcomes was mediated through its effect on blood pressure.

†The *a priori* assumption was that urinary albumin excretion lies on the causal pathway between blood pressure and vascular outcomes and is not a confounder, however sensitivity analyses including adjustment for urinary albumin excretion were conducted.

Figure S2: Mean blood pressure over follow−up in categories defined by quintiles of baseline measurement

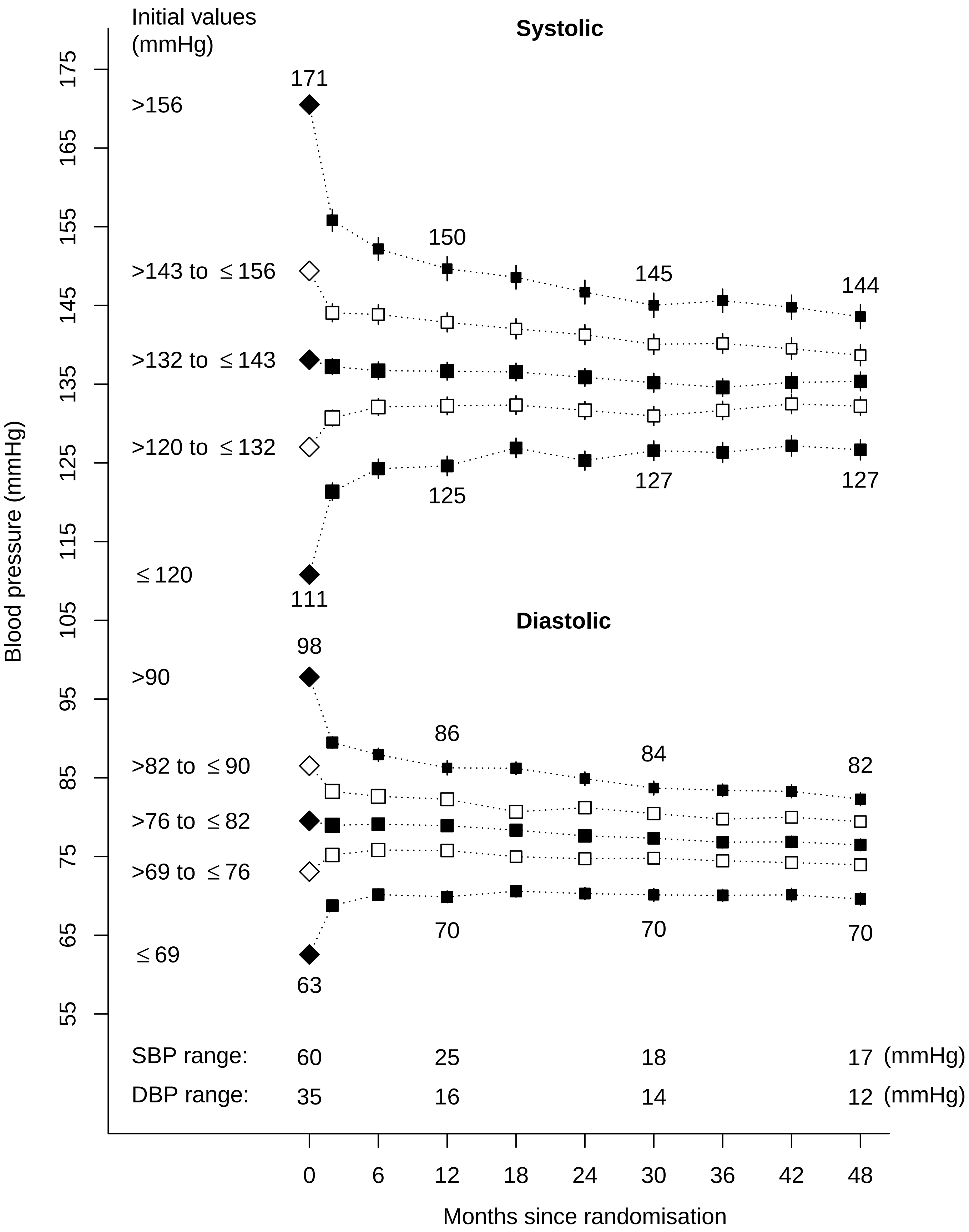

SBP=systolic blood pressure. DBP=diastolic blood pressure. Excludes 4161 participants with missing BP values at any of the follow−up visits.

Figure S3: Association between systolic blood pressure, diastolic blood pressure and pulse pressure and cardiovascular events, subdivided by evidence of prior cardiovascular disease, for those less than 62 years old and 62 years or over

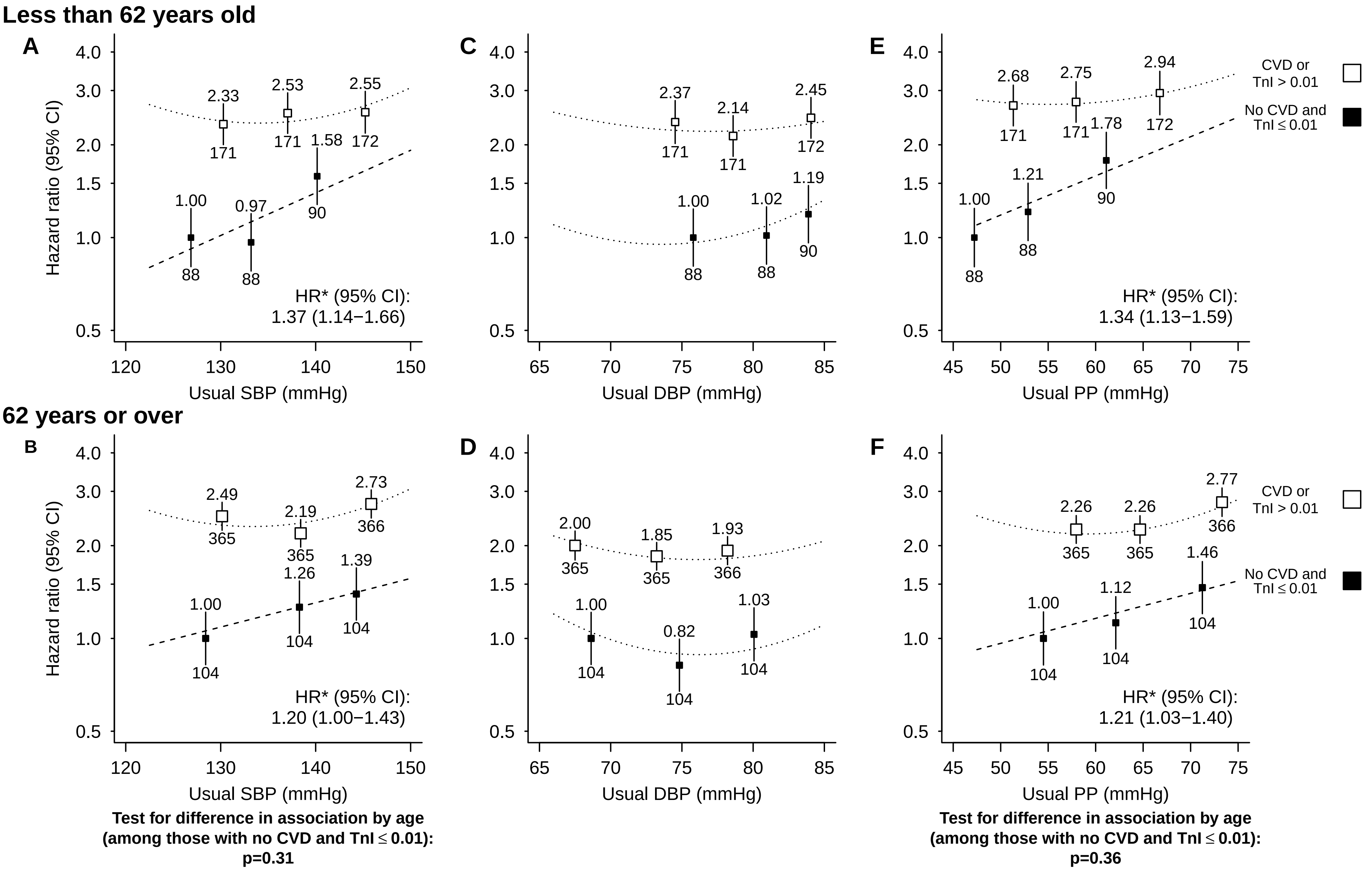

SBP=systolic blood pressure. DBP=diastolic blood pressure. PP=pulse pressure. CVD=self−reported history of cardiovascular disease. TnI=troponin−I (ng/mL). HR=hazard ratio. For each plot, categories of blood pressure contain similar numbers of events. Hazard ratios adjusted for age, sex, ethnicity, country, education, smoking status, prior diabetes, renal replacement therapy status, eGFR, body−mass index and treatment allocation are quoted (above squares) with numbers of events (below). Exclusions as per Table 1. *Hazard ratios per 10 mmHg higher usual SBP/PP are presented for associations where there is no evidence of deviation from a log−linear relationship.

Figure S4: Association between (A) systolic blood pressure, (C) diastolic blood pressure and (E) pulse pressure and non−vascular mortality overall, and association between (B) systolic blood pressure, (E) diastolic blood pressure and (F) pulse pressure and non−vascular mortality subdivided by evidence of prior cardiovascular disease

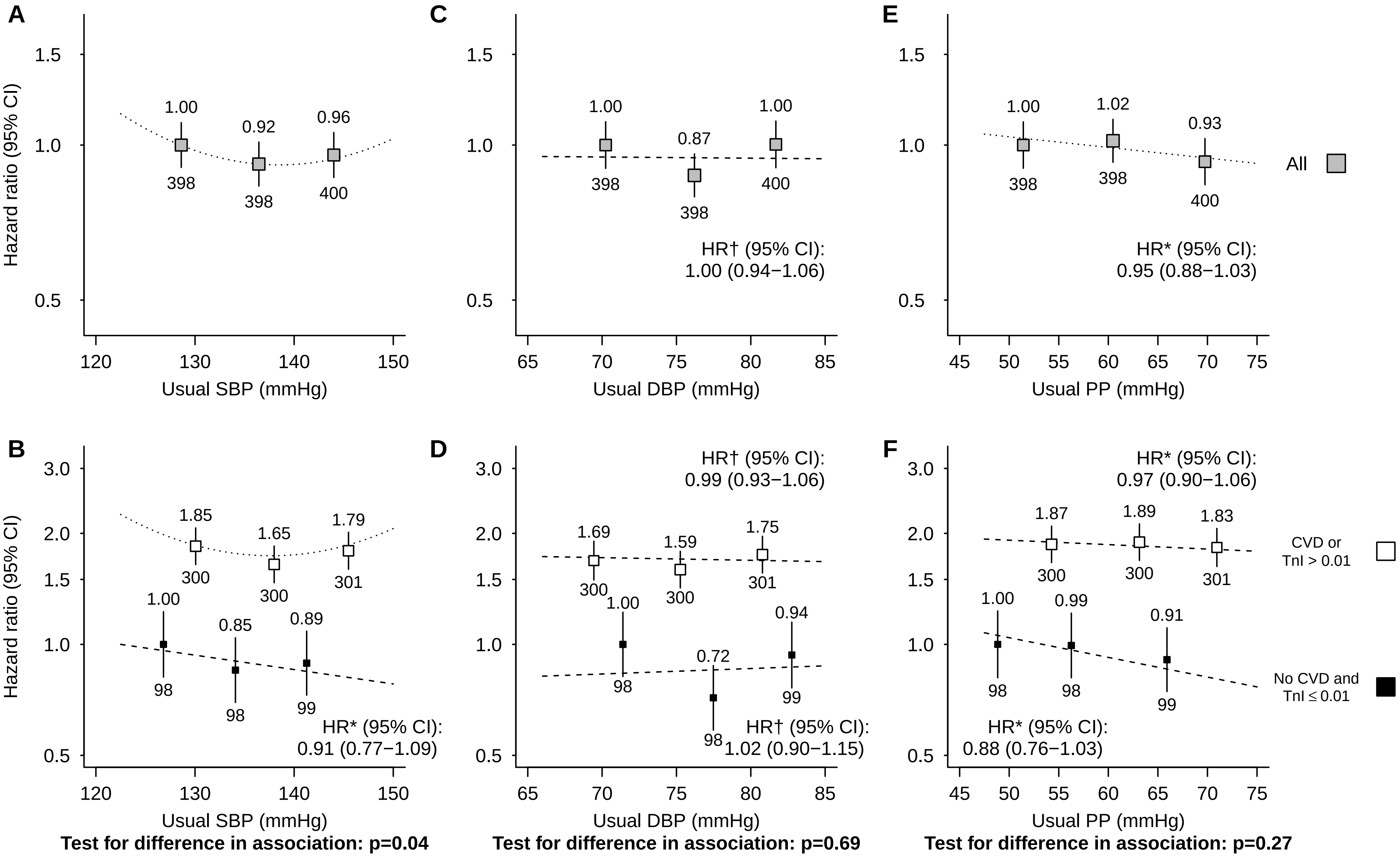

SBP=systolic blood pressure. DBP=diastolic blood pressure. PP=pulse pressure. HR=hazard ratio. CVD=self−reported history of cardiovascular disease. TnI=troponin−I (ng/mL). For each plot, categories of blood pressure contain similar numbers of events. Hazard ratios adjusted for age, sex, ethnicity, country, education, smoking status, prior cardiovascular disease (panels A, C and E only), prior diabetes, renal replacement therapy status, eGFR, body−mass index and treatment allocation are quoted (above squares) with numbers of events (below). Exclusions as per Table 1. *Hazard ratios per 10 mmHg higher usual SBP/PP and †hazard ratios per 5 mmHg higher usual DBP are presented for associations where there is no evidence of deviation from a log−linear relationship.
